# Knowledge attitude, and practice of patients with knee osteoarthritis towards perioperative functional exercise after total knee arthroplasty: a cross-sectional study

**DOI:** 10.7717/peerj.19511

**Published:** 2025-06-04

**Authors:** Houxi Li, Qingqing Su, Lei Qin, Qi Li, Yake Li, Chunyan Wang, Houxia Zhu, Haiyan Li

**Affiliations:** 1 Department of Orthopedics, The Affiliated Hospital of Qingdao University, Qingdao, China; 2 Department of Joint Surgery, Affiliated Hospital of Qingdao University, Qingdao, China; 3 Department of Orthopedic and Joint Surgery, The First Affiliated Hospital of Shandong First Medical University & Shandong Provincial Qianfoshan Hospital, Jinan, China; 4 Qingdao University School of Nursing, Qingdao, China; 5 Lingshui Li Autonomous County People’s Hospital of Hainan Province, Hainan, China

**Keywords:** Knee osteoarthritis, Perioperative functional exercise, Total knee arthroplasty, Cross-sectional study

## Abstract

**Background:**

To investigate the knowledge, attitude, and practice (KAP) in patients with knee osteoarthritis (OA) towards perioperative functional exercise after total knee arthroplasty (TKA).

**Methods:**

The cross-sectional survey enrolled knee OA patients in two tertiary public hospitals at Shandong Province between September 2023 and January 2024. Demographic characteristics, KAP scores, and Tampa Scale for Kinesiophobia (TSK) scores were gathered *via* a self-made questionnaires.

**Results:**

A total of 583 valid questionnaires were collected, 65.01% were females and 48.89% over 65. The average scores of knowledge, attitude, practice and TSK were 11.17 ± 4.31 (possible range: 0–16), 21.78 ± 2.57 (possible range: 6–30), 35.44 ± 5.80 (possible range: 9–45), and 39.21 ± 10.75 (possible range: 17–68), respectively. Spearman correlation analysis showed positive correlation between knowledge and attitude (r = 0.3406, *p* < 0.001), attitude and practice (r = 0.3464, *p* < 0.001), attitude and practice (r = 0.6390, *p* < 0.001), negative correlation between TSK and knowledge (r = −0.3663, *p* < 0.001), attitude (r = −0.2937, *p* < 0.001), and practice (r = −0.3970, *p* < 0.001), respectively. Path analysis found that attitude, underlying diseases, occupation, total knee arthroplasty had direct effects on practice. Knowledge, age, education level, residence, diagnosis time, marital status had indirect effects on practice. Total knee arthroplasty had direct and indirect effects on practice (all *p* < 0.05).

**Conclusions:**

While most patients held a positive attitude and practice, concerns and misconceptions regarding perioperative exercises emerged as urgent issues. It is imperative to implement targeted interventions to enhance adherence by addressing factors influencing KAP.

## Introduction

Knee osteoarthritis (OA) is a prevalent chronic condition characterized as a whole joint disease that affects all joint tissues. While traditionally viewed primarily in terms of articular cartilage degradation, it is now recognized to involve significant subchondral bone remodeling, meniscal degeneration, changes in the infrapatellar fat pad, and inflammation of the synovial membrane (Knights, Redding & Maerz, 2023; Roelofs & De Bari, 2024; Steinmetz et al., 2023). Several well-established risk factors contribute to the development and progression of knee OA. Age is a primary risk factor, with prevalence increasing significantly after 50 years. Sex also plays an important role, with women having a higher prevalence and often experiencing more severe symptoms, particularly after menopause. Obesity represents one of the most significant modifiable risk factors, with each kilogram of weight gain increasing the risk of developing knee OA by 5–10%. Additional risk factors include previous joint trauma, occupations requiring repetitive knee bending or heavy lifting, genetic predisposition, and metabolic factors such as diabetes (Allen & Golightly, 2015; Silverwood et al., 2015). The pathophysiology of KOA involves complex interactions between mechanical, inflammatory, and metabolic factors. Primary KOA develops without a clear initiating cause and is associated with genetic predisposition, aging, obesity, and biomechanical abnormalities, leading to progressive cartilage degeneration, subchondral bone remodeling, and synovial inflammation (Hunter & Bierma-Zeinstra, 2019; Mobasheri & Batt, 2016). Secondary KOA results from identifiable causes such as trauma, congenital joint abnormalities, inflammatory arthritis, or metabolic disorders that alter joint biomechanics and accelerate cartilage breakdown (Hunter & Bierma-Zeinstra, 2019; Mobasheri & Batt, 2016). Total knee arthroplasty (TKA) has become a widely accepted surgical treatment for patients with severe knee OA, aiming to alleviate pain and restore joint function (Altman & Barthel, 2011; Gibbs et al., 2023; Perruccio et al., 2024; Quicke et al., 2022). However, the success of TKA surgery is not solely dependent on the surgical procedure itself; rather, it is also influenced by the patient’s adherence to postoperative rehabilitation protocols, particularly perioperative functional exercise (Bade & Stevens-Lapsley, 2012; Emami et al., 2023; Gränicher et al., 2022; Mo et al., 2023; Witjes et al., 2016).

Functional exercise after TKA plays a crucial role in improving muscle strength, joint stability, and range of motion, which are essential for regaining functional independence and achieving optimal outcomes (Chen et al., 2021; Fortier et al., 2021; Husby et al., 2018; Jones, 2015; Magni, McNair & Rice, 2017). Despite the importance of functional exercise, previous studies have revealed that patients’ recognition and behavior towards it vary significantly (Ferrer-Peña et al., 2021; Nelligan et al., 2020; Zhou et al., 2018). A lack of understanding of the benefits of exercise, negative perceptions about exercise, and poor adherence to exercise protocols have been identified as barriers to successful rehabilitation (Bäcker et al., 2022; Barber-Westin & Noyes, 2016; Harwin et al., 2016).

The KAP (Knowledge, Attitude, and Practice) survey method is a comprehensively utilized structured assessment tool that leverages a questionnaire-based survey to accurately gauge an individual’s comprehension, stance, and practical implementation regarding specific topics. This approach offers a comprehensive understanding of individuals’ perspectives and behaviors towards a given subject matter (Dang et al., 2023; Kotozaki, Norhayati & Nawi, 2021; Tariq et al., 2022), there is no standardized KAP questionnaire for perioperative functional exercise after TKA. Therefore, for this study, we developed and validated a self-designed questionnaire specifically addressing the needs of knee OA patients undergoing TKA.

Given the significant impact of KAP on post-TKA outcomes, it is essential to understand the current status of patients’ KAP towards perioperative functional exercise. This cross-sectional study aimed to investigate the KAP of patients with knee OA towards perioperative functional exercise after TKA. By identifying the gaps in knowledge, negative attitudes, and poor practices, we can develop targeted interventions to improve patients’ adherence to exercise protocols and ultimately enhance their rehabilitation outcomes. The findings of this study are hopeful to provide valuable insights for healthcare professionals in designing and implementing effective rehabilitation programs for patients undergoing TKA.

## Methods

### Patient and public involvement

Patients and/or the public were not involved in the design, conduct, reporting, or dissemination plans of this research.

#### Study design and participants

The cross-sectional survey enrolled knee OA patients in two tertiary public hospitals at Shandong Province between September 2023 and January 2024. The inclusion criteria were: (1) patients diagnosed with knee OA confirmed by clinical physicians; (2) first-time unilateral TKA; (3) clear cognitive function, able to communicate effectively and answer questions. Individuals who were unwilling to participate in this study were excluded. A total of 599 samples were included in this cross-sectional study. The following cases were excluded: (1) 13 questionnaires with answering time less than 60 seconds; (2) 1 questionnaire with age less than 18 years old; (3) 2 questionnaires with abnormal answers to question 10 of basic information. After these exclusions, the final valid dataset comprised 583 cases.

Prior to the commencement of the study, ethical approval was granted by the Ethics Committee of Qingdao University Affiliated Hospital (QYFY WZLL 27989), and informed consent was obtained from all participating individuals.

#### Questionnaire introduction

The questionnaire design was revised based on the suggestions provided by 15 experts, including five orthopedic surgeons, two rehabilitation specialists, three rehabilitation therapists, and five clinical nursing specialists in orthopedic surgery, and underwent a small-sample pilot test (31 copies). The reliability was assessed using Cronbach’s alpha coefficient, which was 0.859, indicating good internal consistency.

The final questionnaire, crafted in Chinese, encompassed a comprehensive data collection spanning five distinct dimensions, comprising a total of 50 items. These dimensions included 10 items for basic information, eight items assessing knowledge, six items evaluating attitudes, nine items focusing on practical application, and 17 items utilizing the Tampa Scale for Kinesiophobia (TSK) for anxiety related to movement. The full questionnaire has been translated into English and is available as Supplemental Material.

Firstly, within the knowledge dimension, each question offered two points for a correct answer, one point for an unclear response, and 0 point for an incorrect answer, resulting in a potential score range of 0 to 16 points. Secondly, for the attitude dimension, a five-point Likert scale was employed, ranging from very positive (five points) to very negative (one point), providing a potential score range of 6 to 30 points. Specifically, questions 1, 3, 4, and 6 adhered to the scoring scheme of a = 5, b = 4, c = 3, d = 2, e = 1, while questions 2 and 5 were reversed, with a = 1, b = 2, c = 3, d = 4, e = 5. Furthermore, the practice dimension encompassed nine questions, also utilizing a five-point Likert scale ranging from always (five points) to never (one point), based on the extent of positive behavior. This resulted in a potential score range of 9 to 45 points. Specifically, questions 1, 2, 3, 5, 7, 8, and 9 followed the scoring pattern of a = 5, b = 4, c = 3, d = 2, e = 1, while questions 4 and 6 were reversed, scoring a = 1, b = 2, c = 3, d = 4, e = 5. At last, the TSK assessment utilized a scoring system of a = 1, b = 2, c = 3, d = 4, except the questions 4, 8, 11, 12 were reversed with scoring a = 4, b = 3, c = 2, d = 1, resulting in a total score range of 17–68. A higher score represents a more severe degree of fear of movement for the patient, and a total score exceeding 37 points indicates a high level of fear of movement.

Questionnaire surveys were administered between the third day post-total knee arthroplasty and the patient’s discharge date. The timing of survey administration within this period was determined based on each individual’s postoperative recovery status, as assessed by the designated nurse. Surveys were conducted using a QR code scanning approach, allowing patients to complete the questionnaire on their personal mobile devices when their condition permitted. This flexible timing approach was designed to ensure patient comfort while obtaining reliable responses, without compromising data validity. For elderly patients with advanced age and impaired vision, the survey was conducted in an interview format, facilitated by the responsible nurse. To guarantee the uniformity and accuracy of the survey, all nurses involved underwent rigorous standardized training, ensuring a consistent approach in data collection and evaluation.

#### Statistical analysis

Statistical analysis was performed using STATA 17.0 (StataCorp LLC, College Station, TX, USA). Quantitative variables were described using Mean ± SD. For inter-group comparisons, the Shapiro-Wilk test was used to assess normality of data distribution. For data that followed a normal distribution, one-way analysis of variance (ANOVA) was used with Bonferroni *post hoc* test for multiple comparisons; for those that did not follow a normal distribution, non-parametric tests (Kruskal-Wallis with Mann-Whitney U test for *post hoc* comparisons) were employed. Categorical variables were described using frequency (percentage). Spearman correlation analysis was used to assess the correlation of knowledge, attitude, practice and TSK. Employing the KAP theoretical framework, we rigorously employed path analysis to validate the intricate interplay between various factors (He et al., 2023; Jiang et al., 2024; Liao & Yang, 2023). We systematically calculated and contrasted both the direct and indirect effects to gain deeper insights (Qin et al., 2019; Singaram et al., 2022). Ensuring the robustness of our model, we imposed stringent goodness-of-fit indices: RMSEA and SRMR were set below 0.08, while TLI and CFI exceeded 0.8 (Suhr, 2006; Ullman, 2006). A *p*-value below 0.05 was deemed statistically significant.

## Results

### Demographic characteristics

Among the 583 participants, nearly two-thirds (65.01%) were females, with 51.11% being under 65 years old and the remaining 48.89% being 65 years old or older. Furthermore, 64.84% hailed from rural or suburban areas, while the rest originated from urban areas. In terms of education, a significant majority (59.69%) had completed junior high school or below, 24.19% had attained a high school or technical school level, and a notable 16.12% had achieved a college, university, or higher degree. Detailed information was showed in Table 1.

**Table 1:** Demographic characteristics.

*N* = 583	*N* (%)	Knowledge		Attitude		Practice	
		Mean ± SD	*P*	Mean ± SD	*P*	Mean ± SD	*P*
**Total score**		11.17 ± 4.31		21.78 ± 2.57		35.44 ± 5.80	
**Gender**			0.779		0.066		0.062
Male	204 (34.99)	11.30 ± 4.12		21.5 ± 2.42		34.85 ± 5.75	
Female	379 (65.01)	11.10 ± 4.41		21.92 ± 2.63		35.74 ± 5.79	
**Age**	60.48 ± 12.78		0.127		**<0.001**		**0.001**
<65	298 (51.11)	11.45 ± 4.22		21.37 ± 2.51		34.59 ± 5.89	
≥65	285 (48.89)	10.87 ± 4.39		22.19 ± 2.56		36.31 ± 5.56	
**Residence type**			0.065		**0.006**		0.088
Rural/Suburban	378 (64.84)	10.93 ± 4.42		21.58 ± 2.54		35.17 ± 5.62	
Urban	205 (35.16)	11.62 ± 4.08		22.12 ± 2.59		35.92 ± 6.08	
**Education level**			**<0.001**		0.094		0.543
Junior high school or below	348 (59.69)	10.78 ± 4.41		21.83 ± 2.56		35.68 ± 5.70	
High school/Technical school	141 (24.19)	11.04 ± 3.93		21.37 ± 2.57		35.08 ± 5.55	
College/University or above	94 (16.12)	12.81 ± 4.13		22.15 ± 2.55		35.03 ± 6.44	
**Your occupation:**			**<0.001**		**<0.001**		**<0.001**
Working	322 (55.23)	11.75 ± 4.18		21.59 ± 2.57		34.81 ± 5.74	
Retired	141 (24.19)	10.97 ± 3.83		22.71 ± 2.47		38.08 ± 5.19	
Unemployed	71 (12.18)	8.816 ± 4.73		20.81 ± 2.33		33.11 ± 5.12	
Other (please specify)	49 (8.4)	11.38 ± 4.71		21.65 ± 2.42		35.28 ± 6.15	
**In the past year, the monthly *per capita* income of your family was (including income in kind and rental income, *etc*.)**: ______ yuan			**<0.001**		0.269		0.122
<2,000	164 (28.13)	10.65 ± 4.34		21.89 ± 2.52		36.01 ± 5.61	
2,000–5,000	242 (41.51)	10.68 ± 4.44		21.57 ± 2.51		34.78 ± 5.73	
5,000–10,000	127 (21.78)	12.41 ± 3.87		21.86 ± 2.65		35.81 ± 6.18	
>10,000	50 (8.58)	12.10 ± 3.96		22.14 ± 2.75		35.70 ± 5.51	
**Your marital status:**			**0.001**		0.179		**0.014**
Single	86 (14.75)	9.662 ± 4.64		21.36 ± 2.14		34.10 ± 5.29	
Married	497 (85.25)	11.43 ± 4.20		21.84 ± 2.63		35.66 ± 5.85	
**Do you have any underlying diseases?**			0.510		**<0.001**		**<0.001**
Diabetes	55 (9.43)	11.03 ± 4.25		22.47 ± 2.82		37.41 ± 5.36	
Hypertension	185 (31.73)	11.00 ± 4.23		22 ± 2.77		36.24 ± 5.92	
Coronary heart disease	169 (28.99)	11.03 ± 4.37		21.03 ± 2.06		33.72 ± 5.49	
Other	174 (29.85)	11.53 ± 4.37		22.04 ± 2.57		35.60 ± 5.69	
**Type of medical insurance:**			0.358		0.152		0.184
Only social medical insurance (*e.g*., employee medical insurance, “new rural cooperative medical care,” “urban residents’ basic medical insurance,” *etc*.)	499 (85.59)	11.24 ± 4.26		21.82 ± 2.51		35.51 ± 5.73	
Only commercial medical insurance	9 (1.54)	11.88 ± 3.29		19.77 ± 3.23		33.55 ± 5.70	
Both social medical insurance and commercial medical insurance	63 (10.81)	10.93 ± 4.60		21.79 ± 2.82		35.80 ± 5.69	
No insurance	12 (2.06)	8.833 ± 5.28		21.08 ± 2.46		31.41 ± 7.70	
**When were you diagnosed with knee osteoarthritis?**			**0.001**		0.088		0.084
Within 1 year	151 (25.9)	11.98 ± 4.59		21.43 ± 2.30		35.01 ± 5.55	
2–3 years	118 (20.24)	11.79 ± 4.20		21.62 ± 2.54		34.83 ± 6.16	
3–5 years	117 (20.07)	10.84 ± 3.96		21.74 ± 2.65		35.27 ± 5.63	
5–10 years	93 (15.95)	10.53 ± 3.89		22.60 ± 2.90		37.03 ± 5.80	
10 years and above	104 (17.84)	10.23 ± 4.50		21.75 ± 2.43		35.48 ± 5.71	
**Have you undergone total knee arthroplasty?**			**0.020**		**0.001**		**<0.001**
Yes	340 (58.32)	11.65 ± 3.80		22.10 ± 2.60		36.52 ± 5.48	
No	243 (41.68)	10.49 ± 4.86		21.31 ± 2.45		33.91 ± 5.88	
**TSK**	39.21 ± 10.75						

**Note:**

Bold values indicate statistical significance (*P* < 0.05).

### Knowledge, attitude, practice and TSK scores

As shown in Table 1, the average scores of knowledge, attitude, practice and TSK were 11.17 ± 4.31 (possible range: 0–16), 21.78 ± 2.57 (possible range: 6–30), 35.44 ± 5.80 (possible range: 9–45), and 39.21 ± 10.75 (possible range: 17–68). Generally, significant disparities in the scores of the knowledge section were observed among individuals with varying education levels (*p* < 0.001), those who were currently employed or not (*p* < 0.001), those with differing monthly *per capita* incomes (*p* < 0.001), individuals with diverse marital statuses (*p* < 0.01), those enduring varying durations of illness (*p* < 0.01), and those who have undergone TKA or not (*p* < 0.05). Furthermore, the study revealed notable disparities in attitude scores among participants belonging to various age brackets (*p* < 0.001), residing in different geographical areas (*p* < 0.01), having distinct occupational statuses (*p* < 0.001), suffering from varied underlying medical conditions (*p* < 0.001), and those who had undergone total knee arthroplasty (TKA) compared to those who had not (*p* < 0.01). In addition, factors such as age group (*p* < 0.01), occupational status (*p* < 0.001), marital status (*p* < 0.05), underlying diseases (*p* < 0.001), and whether or not an individual has undergone TKA surgery (*p* < 0.001) may contribute to variations in scores related to practical aspects.

Specifically, in knowledge dimension (Table S1, Knowledge), the highest score was observed for the item about the understanding of the purpose of medical staff applying effective measures to control pain (K7), with an accuracy of 56.26%. In the attitude dimension (Table S1, Attitude), the item with the highest support rate was about the importance of early postoperative functional exercise after TKA (A1), with 51.63% of participants choosing “strongly agree” and 37.43% choosing “agree”. In the practice dimension (Table S1, practice), the highest score was observed for the item about the communication with medical staff (P8), with 49.4% choosing “Always” and 32.59% choosing “Frequently”.

The average score of TSK was 39.21 ± 10.75, indicating a high level of fear of exercise among the patients (Table 1 and Table S1, TSK). Many participants expressed concerns and fears about the potential pain that may result from exercise, worrying that exercising during the perioperative period may cause them to be injured again. For example, 42.54% (choosing “Strongly Agree” and “Agree”) of the participants were afraid that they would injure themselves if they exercised at this time (TSK 1), 48.92% (choosing “Strongly Agree” and “Agree”) of them were worrying about the accidental injury (TSK 9), and 45.63% (choosing “Strongly Agree” and “Agree”) of them chose to avoid exercise to prevent the pain from worsening (TSK 10).

### Spearman correlation analysis

Spearman correlation analysis showed positive correlation between knowledge and attitude (r = 0.3406, *p* < 0.001), attitude and practice (r = 0.3464, *p* < 0.001), attitude and practice (r = 0.6390, *p* < 0.001), negative correlation between TSK and knowledge (r = −0.3663, *p* < 0.001), attitude (r = −0.2937, *p* < 0.001), and practice (r = −0.3970, *p* < 0.001), respectively (Table 2).

**Table 2:** Pearson correlation analysis.

	Knowledge	Attitude	Practice	TSK
Knowledge	1			
Attitude	0.3406 (*P* < 0.001)	1		
Practice	0.3464 (*P* < 0.001)	0.6390 (*P* < 0.001)	1	
TSK	−0.3663 (*P* < 0.001)	−0.2937 (*P* < 0.001)	−0.3970 (*P* < 0.001)	1

### Path analysis

**Figure 1:**
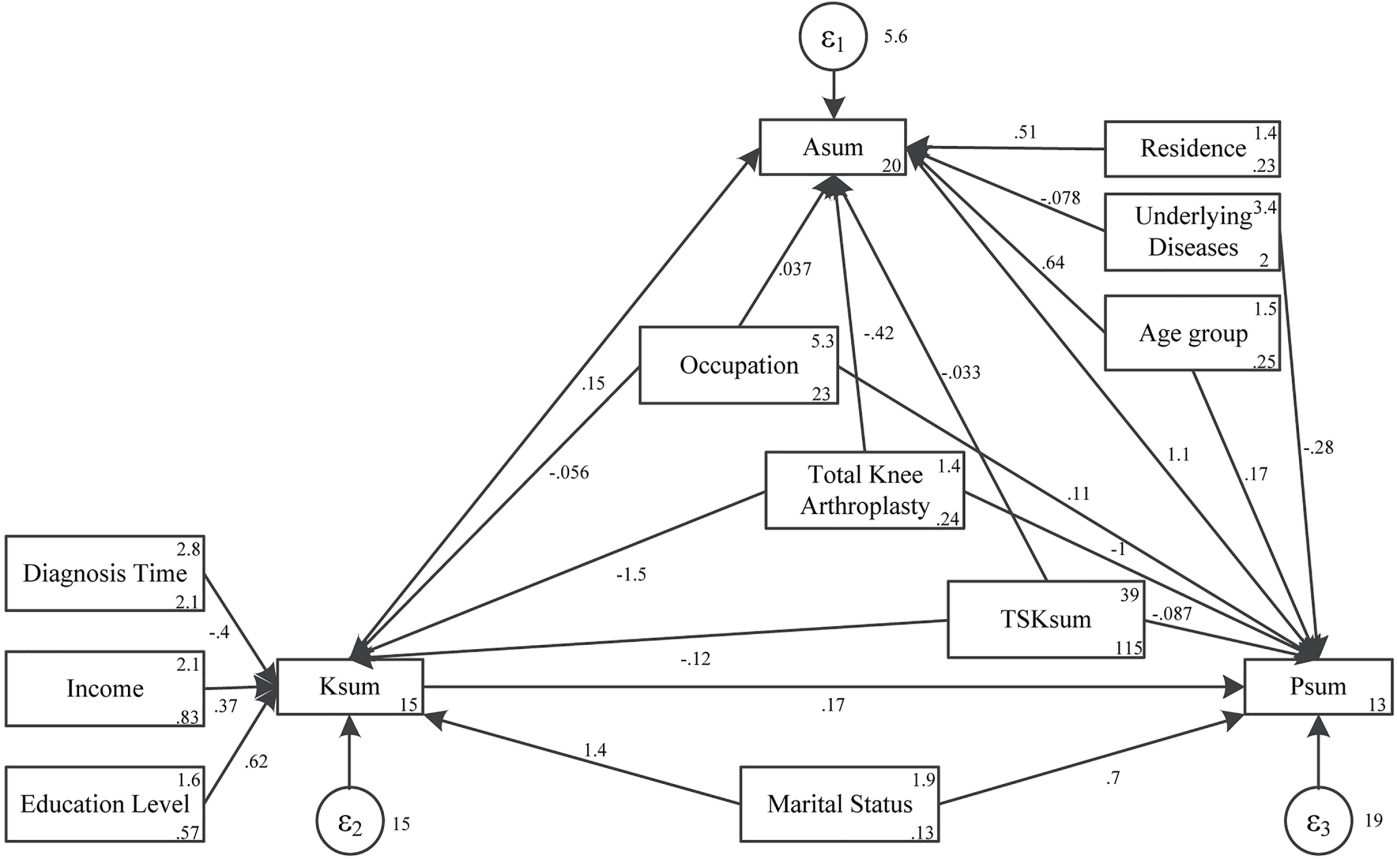
Path analysis.

Path analysis found that education level (β = 0.62, *p* = 0.009), income (β = −0.39, *p* = 0.001), diagnosis time (β = 1.43, *p* = 0.002), underlying diseases (β = −1.46, *p* < 0.001), TSK sum (β = −0.12, *p* < 0.001) had direct effects on knowledge. Knowledge (β = 0.15, *p* < 0.001), age (β = 0.63, *p* = 0.003), residence (β = 0.50, *p* = 0.014) had direct effects on attitude, education (β = 0.09, *p* = 0.016), diagnosis time (β = −0.06, *p* = 0.003) and marital status (β = 0.21, *p* = 0.005) had indirect effects on attitude. Total knee arthroplasty had direct (β = −0.42, *p* = 0.041) and indirect (β = −0.22, *p* < 0.001) effects on attitude. TSK sum had direct (β = −0.03, *p* < 0.001) and indirect (β = −0.01, *p* < 0.001) effects on attitude. Attitude (β = 1.12, *p* < 0.001), underlying diseases (β = −0.28, *p* = 0.027), occupation (β = 0.10, *p* = 0.009), total knee arthroplasty (β = −1.04, *p* = 0.006) had direct effects on practice. Knowledge (β = 0.17, *p* < 0.001), age (β = 0.71, *p* = 0.003), education level (β = 0.21, *p* = 0.016), residence (β = 0.57, *p* = 0.015), diagnosis time (β = −0.13, *p* = 0.003), marital status (β = 0.48, *p* = 0.005) had indirect effects on practice. Total knee arthroplasty had direct (β = −1.04, *p* = 0.006) and indirect (β = −0.97, *p* < 0.001) effects on practice (Table 3 and Fig. 1). Notably, the model fitting indices, including RMSEA (0.000), SRMR (0.010), TLI (1.017), and CFI (1.000), all surpassed the respective threshold values, unequivocally pointing to a good fit (Table S2).

**Table 3:** Path analysis: the total, direct and indirect effects of knowledge, attitude and demographic characteristics on practice.

Model paths		Total effects		Direct effect		Indirect effect	
		β (95% CI)	*P*	β (95% CI)	*P*	β (95% CI)	*P*
A sum<-							
	K sum	0.15 [0.10–0.19]	<0.001	0.15 [0.10–0.19]	<0.001	——	——
	Age	0.63 [0.22–1.05]	0.003	0.63 [0.22–1.05]	0.003	——	——
	Education level	0.09 [0.01–0.17]	0.016	——	——	0.09 [0.01–0.17]	0.016
	Residence	0.50 [0.10–0.91]	0.014	0.50 [0.10–0.91]	0.014	——	——
	Income	0.05 [−0.0 to 0.11]	0.062	——	——	0.05 [−0.0 to 0.11]	0.062
	Diagnosis time	−0.06 [−0.10 to −0.02]	0.003	——	——	−0.06 [−0.10 to −0.02]	0.003
	Marital status	0.21 [0.06–0.36]	0.005	——	——	0.21 [0.06–0.36]	0.005
	Underlying diseases	−0.07 [−0.21 to 0.05]	0.263	−0.07 [−0.21 to 0.05]	0.263	——	——
	Occupation	0.02 [−0.01 to 0.07]	0.195	0.03 [−0.00 to 0.08]	0.086	−0.00 [−0.01 to 0.002]	0.121
	Total knee arthroplasty	−0.64 [−1.05 to −0.22]	<0.001	−0.42 [−0.82 to −0.01]	0.041	−0.22 [−0.34 to −0.09]	<0.001
	TSK sum	−0.05 [−0.07 to −0.03]	<0.001	−0.03 [−0.05 to −0.01]	0.001	−0.01 [−0.02 to −0.01]	<0.001
P sum<-							
	A sum	1.12 [0.97–1.27]	<0.001	1.12 [0.97–1.27]	<0.001	——	——
	K sum	0.33 [0.23–0.44]	0	0.16 [0.07–0.26]	0	0.17 [0.11–0.22]	<0.001
	Age	0.88 [−0.0 to 1.77]	0.053	0.17 [−0.60 to 0.94]	0.663	0.71 [0.23–1.18]	0.003
	Education level	0.21 [0.03–0.38]	0.016	——	——	0.21 [0.03–0.38]	0.016
	Residence	0.57 [0.10–1.03]	0.015	——	——	0.57 [0.10–1.03]	0.015
	Income	0.12 [−0.0 to 0.26]	0.061	——	——	0.12 [−0.0 to 0.26]	0.061
	Diagnosis time	−0.13 [−0.22 to −0.04]	0.003	——	——	−0.13 [−0.22 to −0.04]	0.003
	Marital status	1.18 [0.13–2.23]	0.026	0.69 [−0.30 to 1.70]	0.174	0.48 [0.14–0.82]	0.005
	Underlying diseases	−0.37 [−0.66 to −0.07]	0.013	−0.28 [−0.53 to −0.03]	0.027	−0.08 [−0.24 to 0.06]	0.264
	Occupation	0.12 [0.033–0.22]	0.008	0.10 [0.026–0.18]	0.009	0.023 [−0.03 to 0.07[	0.4
	Total knee arthroplasty	−2.01 [−2.90 to −1.12]	0	−1.04 [−1.78 to −0.30]	0.006	−0.97 [−1.49 to −0.44]	<0.001
	TSK sum	−0.16 [−0.20 to −0.12]	0	−0.08 [−0.12 to −0.05]	0	−0.07 [−0.10 to −0.05]	<0.001
K sum<-							
	Education level	0.62 [0.15–1.09]	0.009	0.62 [0.15–1.09]	0.009	——	——
	Residence	0.37 [−0.0 to 0.75]	0.05	0.37 [−0.0 to 0.75]	0.05	——	——
	Income	−0.39 [−0.63 to −0.16]	0.001	−0.39 [−0.63 to −0.16]	0.001	——	——
	Diagnosis time	1.43 [0.54–2.32]	0.002	1.43 [0.54–2.32]	0.002	——	——
	Marital status	−0.05 [−0.12 to 0.012]	0.109	−0.05 [−0.12 to 0.012]	0.109	——	——
	Underlying diseases	−1.46 [−2.13 to −0.78]	<0.001	−1.46 [−2.13 to −0.78]	<0.001	——	——
	TSK sum	−0.12 [−0.15 to −0.09]	<0.001	−0.12 [−0.15 to −0.09]	<0.001	——	——

## Discussion

This study provides crucial insights into the KAP towards perioperative functional exercise post-TKA among knee OA patients. Despite a generally positive attitude and willingness to exercise, apprehensions and misconceptions about perioperative exercises stand out as urgent issues demanding prompt attention. It is vital to adopt targeted measures to boost patients’ adherence to perioperative exercise by intervening in factors that significantly, directly or indirectly, impact their KAP.

Notably, this study shows that KAP scores differed in patients with different age group, residence, educational level, employment status, income level, and marital status. This suggests that these socio-economic factors play a significant role in shaping patients’ knowledge and attitudes regarding their treatment options and rehabilitation protocols. While our study focused on measuring knowledge and attitudes, these factors likely also influence behavioral outcomes. This connection is supported by a study which is demonstrated that occupational and family factors influence compliance with treatment regimens (Zhou et al., 2018). The relationship between improved knowledge and better compliance is well-established in health behavior models, suggesting that enhancing patient knowledge through targeted education may ultimately improve adherence to rehabilitation protocols. Additionally, the presence of comorbidities such as hypertension, coronary heart disease, and diabetes appears to negatively impact patients’ attitude towards perioperative exercise, likely due to concerns over the additional physical demands.

In terms of knowledge, patients showed a certain degree of understanding of the necessity of perioperative rehabilitation training for TKA but lacked a clear understanding of the critical period of rehabilitation training and the importance of preoperative rehabilitation training. This knowledge gap may be attributed to several factors. First, the current clinical education for TKA patients in our setting predominantly emphasizes postoperative rehabilitation while providing less information about the critical timeframes and preoperative preparation. Second, the complex medical terminology and concepts related to rehabilitation phases may be difficult for patients to comprehend, particularly those with lower educational levels who comprised a significant portion of our sample. Third, information overload during pre-surgical consultations might prevent patients from fully absorbing all details about rehabilitation timing. Currently, patients typically receive this information through pre-surgical consultations, written materials, and brief sessions with rehabilitation specialists, but the effectiveness of these communication methods varies widely and appears insufficient for ensuring comprehensive understanding. Therefore, patient education should start from these two aspects. In the attitude dimension, patients demonstrated a strong sense of agreement regarding the importance of early postoperative functional exercise and the critical role of effective pain control. However, a considerable number of patients expressed fears and concerns about perioperative exercise, particularly related to the fear of re-injuring the knee or not being able to tolerate the intensity of the exercises. These findings highlight the need for targeted interventions to address patients’ fears and anxieties, as well as to provide detailed and tailored rehabilitation guidance. The practice dimension reveals a generally positive engagement in perioperative exercise, with most patients reporting regular communication with medical staff and a willingness to comply with rehabilitation protocols. However, the high levels of fear and anxiety identified in the attitude dimension suggest that this positive engagement may be fragile and dependent on continued support and encouragement from medical professionals. Briefly, while patients generally demonstrated a positive attitude and willingness to engage in exercise, there were still areas of concern and misunderstanding that need to be addressed through targeted interventions and enhanced patient education. By doing so, we can help patients achieve better recovery outcomes and improve their quality-of-life following TKA (Rice et al., 2019; Smith et al., 2017).

Furthermore, the high TSK scores highlights a significant concern among participants regarding the potential pain and injury associated with exercise during the perioperative period. The high percentages of participants expressing fears such as reinjury during exercise, anxiety over accidental harm, and avoidance of movement to prevent pain exacerbation indicated a need for targeted interventions to address these anxieties. Previous studies have already demonstrated similar conclusions (Brown et al., 2016). Güney-Deniz et al. (2017) pointed out that early rehabilitation outcomes after TKA were influenced by pain-related kinesiophobia, while Filardo et al. (2016) demonstrated that kinesiophobia was an independent factor influencing the outcome after TKA independently from other psychological and physical variables. The limited understanding of pain among participants highlighted a gap in patient education. This misperception may hinder patients’ engagement in essential rehabilitation programs, as they may perceive pain as a barrier rather than a natural part of the healing process, which further underscored this need for education on the benefits of appropriate exercise within pain thresholds.

The significant positive correlations between knowledge, attitude, and practice scores, as well as the negative correlations with TSK scores, suggest that enhancing patients’ knowledge regarding the importance and benefits of perioperative exercises may positively influence their attitudes and subsequent practices. Conversely, fear of movement and pain, reflected in higher TSK scores, appears to be a barrier to engagement in recommended exercise programs. Based on these findings, we recommend the implementation of multifaceted educational interventions aimed at addressing patients’ fears and misperceptions about perioperative exercises. Such interventions could include educational sessions led by healthcare professionals, provision of written materials, and the use of digital tools and applications to deliver tailored information and support. Another study delved into the perspectives and experiences of patients with knee OA regarding a self-directed e-health intervention designed to facilitate exercise. The findings suggested that such electronic health interventions have the potential to significantly enhance patients’ rehabilitation training experience and boost their compliance (Nelligan et al., 2020). By improving patients’ knowledge and attitudes towards perioperative exercises, we may be able to reduce their fear of movement, enhance their engagement in rehabilitation programs, and ultimately improve their functional outcomes and quality of life.

Additionally, several noteworthy findings emerge from the SEM analysis. Firstly, knowledge, age, and residence positively correlate with attitude towards perioperative exercises. Specifically, older patients and those residing in urban areas tend to have a more positive attitude towards exercise, likely due to their greater appreciation of the benefits of physical activity and rehabilitation. Conversely, TKA surgery and TSK scores have a negative impact on attitude, indicating that surgical procedures and fear of movement may hinder patients’ willingness to engage in exercises. Secondly, attitude and knowledge positively influence practice, with attitude exerting a more significant effect. This finding underscores the importance of positive attitudes in motivating patients to engage in perioperative exercises. This discovery resonates with a prior study indicating that adding an motor imagery intervention to standard rehabilitation for patients with TKA may improve quadriceps strength and pain intensity (Ferrer-Peña et al., 2021). On the other hand, underlying medical conditions, TKA surgery, and TSK scores have negative impacts on practice, indicating that patients with comorbidities, recent surgical interventions, or high fear of movement may face greater challenges in adhering to rehabilitation protocols. A previous study has also found that undergoing TKA surgery could have a negative impact on patients’ compliance with exercise, possibly due to the patients’ belief that their symptoms have improved after surgery (Zhou et al., 2018). Additionally, education level and marital status positively correlate with knowledge, suggesting that patients with higher educational attainment and social support may be better equipped to understand and appreciate the importance of perioperative exercises. Conversely, diagnosis duration, TKA surgery, and TSK scores negatively correlate with knowledge, indicating that patients with longer disease duration, recent surgical procedures, or high fear of movement may have less understanding of the benefits of rehabilitation. In conclusion, the SEM analysis provides a comprehensive understanding of the factors influencing KAP towards perioperative exercises among OA patients undergoing TKA. The findings highlight the importance of addressing patients’ fears and misperceptions, enhancing their knowledge and attitudes, and ultimately motivating them to engage in rehabilitation programs. Future research and interventions should focus on these areas to improve patients’ adherence to perioperative exercise protocols and enhance their functional outcomes (Meier et al., 2008; Quack et al., 2015; Skoffer, Dalgas & Mechlenburg, 2014; Standifird, Cates & Zhang, 2014).

While this study has yielded invaluable insights, it is crucial to recognize a few limitations. Firstly, the relatively small sample size may hinder the study’s ability to comprehensively represent the broader population of knee OA patients. A larger sample would be beneficial to provide more robust and generalizable results. Secondly, the cross-sectional design restricted the ability to establish causal relationships between patient knowledge and other variables. A more holistic approach, incorporating longitudinal studies, would be instrumental in examining the evolution of patient knowledge over time and its consequential impact on outcomes.

It’s worth noting that different surgical approaches for knee replacement can significantly impact patient outcomes and rehabilitation experiences. While our study focused on TKA patients, unicompartmental knee arthroplasty (UKA) is an alternative option for patients with isolated compartmental disease. Previous research has shown that UKA typically requires less extensive rehabilitation and is associated with faster recovery times compared to TKA (Liddle et al., 2014). Furthermore, the choice between UKA and TKA can influence patients’ post-surgical quality of life, with UKA often demonstrating advantages in terms of proprioception, kneeling ability, and participation in recreational activities (Kleeblad et al., 2018). However, TKA remains advantageous for more severe, multi-compartmental disease and offers excellent long-term durability. Future studies should consider exploring how KAP towards perioperative exercise might differ between UKA and TKA patients, as this could inform more tailored rehabilitation strategies for each surgical approach and potentially enhance overall quality of life outcomes for knee OA patients undergoing joint replacement surgery.

Several limitations should be acknowledged in this study. First, the cross-sectional design precludes establishing casual relationships between variables, and longitudinal research would be valuable to track how KAP evolves throughout the rehabilitation process. Second, self-reported data may be subject to recall bias and social desirability bias, potentially affecting the accuracy of responses. Third, our sample was drawn from only two tertiary hospitals in Shandong Province, which may limit the generalizability of findings to patients in other regions or healthcare settings with different socioeconomic profiles and medical resources. Fourth, while our questionnaire underwent expert validation and reliability testing, a more extensive validation process involving larger samples across different regions would strengthen the measurement tool. Finally, we did not control for the potential influence of comorbidities or pain severity on KAP scores, which could confound some of the relationships observed.

In conclusion, the study offers profound insights into patients’ KAP towards perioperative exercise after TKA among those with knee OA. While positive attitudes and practices prevailed, apprehensions and misconceptions emerged as urgent issues. Targeted measures are needed to boost adherence to perioperative exercise, focusing on factors influencing patients’ KAP.

## Supplemental Information

10.7717/peerj.19511/supp-1 Supplemental Information 1 Data.

10.7717/peerj.19511/supp-2 Supplemental Information 2 Supplementary material.

10.7717/peerj.19511/supp-3 Supplemental Information 3 Questionnaire (Chinese).

10.7717/peerj.19511/supp-4 Supplemental Information 4 Questionnaire (English).

## Additional Information and Declarations

### Data Availability

The following information was supplied regarding data availability:

The raw data is available in the Supplemental File.
