# Supplementary material for: Knowledge attitude, and practice of patients with knee osteoarthritis towards perioperative functional exercise after total knee arthroplasty: a cross-sectional study
Source: PeerJ. 2025 Jun 4;13:e19511. doi: 10.7717/peerj.19511 (PMC12145091; doi:10.7717/peerj.19511)
Supplement: Supplemental Information 2 [file peerj-13-19511-s002.docx]

**Supplementary table 1.** Knowledge, attitude, practice and TSK dimensions.

| **Knowledge** (with correct answers) | | **N (%)** | | | | |  |
| --- | --- | --- | --- | --- | --- | --- | --- |
|  |  | **Correct** | | **Incorrect** | | **Unclear** | |
| 1. **Through joint replacement, the pain and discomfort caused by degeneration of knee joint cartilage can be relieved. What is the key period for the recovery of knee joint function?**   The early postoperative period, especially within the first half month after surgery. | | 255 (43.74) | | 249 (42.71) | | 79 (13.55) | |
| 1. **How well do you understand the benefits of active functional exercises before surgery?**   Preoperative active functional exercises can increase muscle strength, alleviate postoperative pain, and shorten the recovery time after surgery. | | 255 (43.74) | | 242 (41.51) | | 51 (8.75) | |
| 1. **Are you aware of the benefits of early postoperative joint activity and functional exercises?**   Early postoperative joint activity and functional exercises can effectively reduce the occurrence of perioperative complications, facilitate the recovery of knee joint function and overall physical fitness. | | 290 (49.74) | | 225 (38.59) | | 44 (7.55) | |
| 1. **Do you understand the purposes of rehabilitation exercises after knee joint replacement surgery?**   To improve and maintain normal joint mobility, enhance knee muscle strength, promote knee joint stability; to master joint protection techniques, avoid excessive pressure on the joint prosthesis, and extend the service life of the artificial joint as much as possible. | | 314 (53.86) | | 219 (37.56) | | 47 (8.06) | |
| 1. **Do you know what rehabilitation exercises can be performed from the recovery of anesthesia after knee joint replacement surgery?**   You can perform muscle and distal joint rehabilitation exercises in bed, and on the first day after surgery, you can do knee joint flexion and extension exercises and get out of bed under the guidance of medical staff. | | 317 (54.37) | | 235 (40.31) | | 57 (9.78) | |
| 1. **Do you know how to perform functional exercises in bed?** | | 291 (49.91) | | 201 (34.48) | | 54 (9.26) | |
| 1. **Are you aware of the purpose of medical staff applying effective measures (painkillers, local cold compress, etc.) to control pain after surgery?**   To reduce pain and actively engage in rehabilitation training under the guidance of medical staff. | | 328 (56.26) | | 207 (35.51) | | 70 (12.01) | |
| 1. **Can rehabilitation exercises during the perioperative period of total knee arthroplasty be performed only on the affected limb?**   It is not just about exercising the affected limb, but should also include training for both upper limbs, the healthy side lower limb, and the affected limb. | | 306 (52.49) | | 201 (34.48) | | 115 (19.73) | |
| **Attitude** | **Strongly Agree** | **Agree** | **Neutral** | | **Disagree** | **Strongly Disagree** | |
| 1. I believe that early postoperative functional exercise after total knee arthroplasty is crucial for recovery and can significantly improve my quality of life. | 301 (51.63) | 220 (37.74) | 57 (9.78) | | 2 (0.34) | 3 (0.51) | |
| 1. I consider it very dangerous to start functional exercises on the day of surgery, and I am very concerned even about activities in bed. | 146 (25.04) | 202 (34.65) | 118 (20.24) | | 73 (12.52) | 44 (7.55) | |
| 1. I realize the importance of postoperative functional exercise for recovery, and I will strive to achieve the rehabilitation goals set before surgery. | 277 (47.51) | 219 (37.56) | 76 (13.04) | | 5 (0.86) | 6 (1.03) | |
| 1. I do not understand the postoperative rehabilitation exercises for knee arthroplasty well and hope for more detailed guidance and explanation. | 261 (44.77) | 200 (34.31) | 89 (15.27) | | 24 (4.12) | 9 (1.54) | |
| 1. Perioperative functional exercise for total knee arthroplasty involves not only the affected limb but also other parts. I am concerned about whether I can adapt to this exercise method. | 178 (30.53) | 228 (39.11) | 110 (18.87) | | 50 (8.58) | 17 (2.92) | |
| 1. I understand that effective pain control is key to rehabilitation exercise. I will actively communicate with medical staff to ensure that the pain is effectively relieved. | 286 (49.06) | 225 (38.59) | 63 (10.81) | | 6 (1.03) | 3 (0.51) | |
| **Practice** | **Always** | **Frequently** | **Sometimes** | | **Rarely** | **Never** | |
| 1. I will proactively seek knowledge about postoperative rehabilitation after total knee arthroplasty. | 234 (40.14) | 209 (35.85) | 108 (18.52) | | 27 (4.63) | 5 (0.86) | |
| 1. A slight discomfort during early exercise is normal, and I will continue to persevere. However, if there is severe pain, I will seek medical attention promptly. | 237 (40.65) | 206 (35.33) | 116 (19.9) | | 20 (3.43) | 4 (0.69) | |
| 1. During the recovery period after surgery, I will actively participate in functional exercises guided by medical staff to promote knee joint recovery. | 271 (46.48) | 203 (34.82) | 85 (14.58) | | 21 (3.6) | 3 (0.51) | |
| 1. Due to fear, I may avoid or reduce participation in rehabilitation exercises. | 97 (16.64) | 113 (19.38) | 163 (27.96) | | 113 (19.38) | 97 (16.64) | |
| 1. In order to improve knee joint mobility and muscle strength, I will actively engage in functional exercises as recommended by my doctor after surgery. | 266 (45.63) | 214 (36.71) | 83 (14.24) | | 19 (3.26) | 1 (0.17) | |
| 1. I am worried about injuring my knee joint again and am unwilling to engage in more vigorous exercises even if recommended by the doctor. | 104 (17.84) | 132 (22.64) | 127 (21.78) | | 116 (19.9) | 104 (17.84) | |
| 1. I will actively participate in rehabilitation exercises to improve my quality of life, reduce joint pain, and better perform daily activities. | 278 (47.68) | 197 (33.79) | 87 (14.92) | | 18 (3.09) | 3 (0.51) | |
| 1. I will actively communicate with medical staff. Their support is vital for rehabilitation exercises, and their assistance will give me more confidence in coping with the recovery process. | 288 (49.4) | 190 (32.59) | 88 (15.09) | | 15 (2.57) | 2 (0.34) | |
| 1. I will actively seek guidance for rehabilitation exercises to ensure correct movements and avoid improper actions that may cause injury. | 234 (40.14) | 209 (35.85) | 108 (18.52) | | 27 (4.63) | 5 (0.86) | |
| **TSK** | **Stronly Disagree** | **Disagree** | **Agree** | | **Stronly Agree** |  |  |
| 1. I am afraid that I will injure myself if I exercise at this time. | 130 (22.3) | 205 (35.16) | 215 (36.88) | | 33 (5.66) |  |  |
| 1. If I try to overcome my fear, the pain will intensify. | 146 (25.04) | 213 (36.54) | 192 (32.93) | | 32 (5.49) |  |  |
| 1. My body tells me that I have made a very serious mistake. | 146 (25.04) | 265 (45.45) | 142 (24.36) | | 30 (5.15) |  |  |
| 1. If I exercise at this time, the pain is likely to be relieved. | 106 (18.18) | 192 (32.93) | 242 (41.51) | | 43 (7.38) |  |  |
| 1. People around me do not pay enough attention to my health condition. | 228 (39.11) | 249 (42.71) | 86 (14.75) | | 20 (3.43) |  |  |
| 1. Unexpected events will keep my body at risk. | 124 (21.27) | 185 (31.73) | 230 (39.45) | | 44 (7.55) |  |  |
| 1. Pain always means that the body is being injured from the outside. | 129 (22.13) | 203 (34.82) | 216 (37.05) | | 35 (6) |  |  |
| 1. Exercise that only worsens the pain does not necessarily mean it is dangerous. | 119 (20.41) | 195 (33.45) | 232 (39.79) | | 37 (6.35) |  |  |
| 1. I am afraid of accidentally injuring myself. | 125 (21.44) | 167 (28.64) | 253 (43.4) | | 38 (6.52) |  |  |
| 1. The safest thing I can do now to prevent the pain from worsening is to avoid excessive exercise and be cautious in everything I do. | 133 (22.81) | 184 (31.56) | 229 (39.28) | | 37 (6.35) |  |  |
| 1. If I don't have any underlying risk factors, I wouldn't feel such pain. | 106 (18.18) | 155 (26.59) | 279 (47.86) | | 43 (7.38) |  |  |
| 1. I am in a lot of pain, but if I exercise actively, this situation will improve. | 92 (15.78) | 147 (25.21) | 284 (48.71) | | 60 (10.29) |  |  |
| 1. Pain tells me when to stop exercising to prevent injury. | 80 (13.72) | 132 (22.64) | 323 (55.4) | | 48 (8.23) |  |  |
| 1. Being as active as I am really unsafe. | 136 (23.33) | 241 (41.34) | 172 (29.5) | | 34 (5.83) |  |  |
| 1. I am too prone to injury to do what ordinary people can do. | 144 (24.7) | 252 (43.22) | 156 (26.76) | | 31 (5.32) |  |  |
| 1. Although some things cause me pain, I don't think they are dangerous. | 89 (15.27) | 171 (29.33) | 275 (47.17) | | 48 (8.23) |  |  |
| 1. No one has to exercise when they are in pain. | 89 (15.27) | 147 (25.21) | 289 (49.57) | | 58 (9.95) |  |  |

**Supplementary table 2.** Model fit.

| **Indicators** | **Reference** | **Results** |
| --- | --- | --- |
| RMSEA | < 0.08 Good | 0.000 |
| SRMR | < 0.08 Good | 0.010 |
| TLI | > 0.8 Good | 1.017 |
| CFI | > 0.8 Good | 1.000 |
