# Supplementary material for: Knowledge attitude, and practice of patients with knee osteoarthritis towards perioperative functional exercise after total knee arthroplasty: a cross-sectional study
Source: PeerJ. 2025 Jun 4;13:e19511. doi: 10.7717/peerj.19511 (PMC12145091; doi:10.7717/peerj.19511)
Supplement: Supplemental Information 3 [file peerj-13-19511-s003.docx]

| 问卷编号： |
| --- |
| 亲爱的参与者：  我们是青岛大学附属医院关节外科，诚挚邀请您参与我们的课题研究。本研究旨在了解**膝骨性关节炎患者对全膝关节置换围术期功能锻炼的知识、态度和实践**，为制定科学的干预策略提供依据，这可能在未来帮助到更多的人，改善他们的健康情况。您参与这项研究是自愿的，本研究已通过伦理审查委员会的审查，如果您同意参与本研究，请参阅以下说明。  1.请您完成问卷，答案无关对错，您只需要根据实际情况填写。回答过程中的任何问题您都可以向我们提出，完成后，请您及时提交。  2.本研究是简单的问卷调查，不会对您的身体和心理状况造成伤害，但会涉及一些隐私问题，如您的性别、年龄等，我们会严格保密，不会泄露您的信息，请您放心填写。  3.作为参与者，您可以随时了解与本研究相关的信息和研究进展，如果您决定退出研究，请告知我们，您的数据将不包含在研究结果中。  最后，衷心感谢您能百忙之中抽出时间支持我们的科学研究！  □我已知晓并同意将所收集的数据用于科学研究。  知情同意签字：  参与时间： 年 月 日 |

| **第一部分 基本信息** | | |
| --- | --- | --- |
| **1.您的性别：** | a.男 | b.女 |
| **2.您的年龄： 。**（仅填数字，问卷星中麻烦设置数字验证-整数） | | |
| **3.您的居住地：** | a.农村  b.城市  c.城郊 | |
| **4.您的教育程度：** | a.初中及以下  b.高中/中专  c.大专  d.本科  e.硕士及以上 | |
| **5.您的职业类型：** | a.政府/机关干部/公务员  b.专业技术人员（教师、医生、工程技术人员、作家等专业人员）  c.普通职员和有关人员  d.普通工人（工厂工人/体力劳动者）  e.商业、服务业人员  f.个体经营者/承包商  g.自由职业  h.农、林、牧、渔水利生产人员  I生产、运输设备操作人员及有关人员  J退休  K暂无职业  L其他（请注明） | |
| **6.过去一年，您家每月人均收入为(包含实物收入和租房收入等):______元** | a.<2000  b.2000-5000  c.5000-10000  d.10000-20000  e.>20000 | |
| **7.您的婚姻情况：** | a.未婚  b.已婚  c.离异  d.丧偶 | |
| **8.您是否有基础疾病？（多选）** | a.糖尿病  b.高血压  c.肾脏病  d.其他 | |
| **9.医保类型：** | a.仅有社会医疗保险（如：职工医保、“新农合”、“城居保”等）  b.仅有商业医疗保险  c.既有社会医疗保险又有商业医疗保险  d.无保险 | |
| **9.您何时确诊的膝骨性关节炎？（请选择时间）** | | |
| **10.您是否进行过全膝关节置换？** | a.是  b.否 | |

| **第二部分 对全膝关节置换围术期功能锻炼相关知识的知晓情况** | | | |  |
| --- | --- | --- | --- | --- |
| **下列陈述，您了解的程度是：** | | | | |
| **1.通过关节置换可以缓解因膝关节软骨退变导致的疼痛不适，膝关节功能恢复的关键时期是什么时候？**  术后早期，特别是手术后半个月内。 | a.很了解 | b.听说过 | c.不清楚 | |
| **2.您对于手术前积极功能锻炼的好处有多了解？**  术前积极功能锻炼可以增加肌肉力量，减轻术后疼痛，缩短术后恢复时间。 | a.很了解 | b.听说过 | c.不清楚 | |
| **3.您是否知晓术后早期关节活动和功能锻炼的好处？**  术后早期关节活动和功能锻炼能有效减少围手术期并发症的发生，利于膝关节功能及全身体力的康复。 | a.很了解 | b.听说过 | c.不清楚 | |
| **4.您是否了解膝关节置换术后康复锻炼的目的？**  改善、维持正常关节活动度，增强膝部肌力，促进膝关节稳定性；掌握关节保护技巧，避免对关节假体施加过多压力，尽可能延长人工关节的使用期限。 | a.很了解 | b.听说过 | c.不清楚 | |
| **5.您是否了解自膝关节置换术后麻醉恢复起，可以进行哪些康复锻炼？**  可在床上行肌肉与远端关节的康复锻炼，术后第1天经医护人员指导后可行膝关节伸屈及下床练习。 | a.很了解 | b.听说过 | c.不清楚 | |
| **6.您是否知道如何进行床上功能锻炼？** | a.很了解 | b.听说过 | c.不清楚 | |
| **7. 您是否了解医护人员术后应用有效措施（止痛药物、局部冷敷等）控制疼痛的目的？**  减少疼痛，配合医护人员主动进行康复训练。 | a.很了解 | b.听说过 | c.不清楚 | |
| **8.全膝关节置换围术期功能锻炼仅锻炼患肢就可以吗？**  不只是锻炼患肢，应包括双上肢、健侧下肢和患肢的训练。 | a.很了解 | b.听说过 | c.不清楚 | |

| **第三部分 对全膝关节置换围术期功能锻炼的态度想法** | | | | | |
| --- | --- | --- | --- | --- | --- |
| **1.我相信全膝关节置换手术的术后早期的功能锻炼对恢复至关重要，可以显著改善我的生活质量。** | a.非常同意 | b.同意 | c.一般 | d.不同意 | e.非常不同意 |
| **2.我认为手术当天就开始功能锻炼是很危险的，即使是在床上活动我也非常担心。** | a.非常同意 | b.同意 | c.一般 | d.不同意 | e.非常不同意 |
| **3.我意识到术后的功能锻炼对于术后恢复的重要性，我会努力达到术前设定的康复目标。** | a.非常同意 | b.同意 | c.一般 | d.不同意 | e.非常不同意 |
| **4.我对于膝关节置换术后的康复锻炼不太了解，希望能有更详细的指导和解释。** | a.非常同意 | b.同意 | c.一般 | d.不同意 | e.非常不同意 |
| **5.全膝关节置换围术期功能锻炼不仅涉及患肢，还包括其他部位，我担心是否能适应这样的锻炼方式。** | a.非常同意 | b.同意 | c.一般 | d.不同意 | e.非常不同意 |
| **6.我理解良好的疼痛控制是康复锻炼的关键，我会积极与医护人员沟通，确保疼痛得到有效缓解。** | a.非常同意 | b.同意 | c.一般 | d.不同意 | e.非常不同意 |

| **第四部分 恐动症TSK评分量表** | | | | |
| --- | --- | --- | --- | --- |
| **1.此时如果我运动的话，我害怕会伤害到我自己** | a.完全反对 | b.反对 | c.同意 | d.完全同意 |
| **2.如果我尝试克服恐惧，疼痛会加剧** | a.完全反对 | b.反对 | c.同意 | d.完全同意 |
| **3.我的身体告诉自己我犯了非常严重的错误** | a.完全反对 | b.反对 | c.同意 | d.完全同意 |
| **4.此时如果我运动的话疼痛很可能会有所缓解** | a.完全反对 | b.反对 | c.同意 | d.完全同意 |
| **5.周围人不够关注我的健康状况** | a.完全反对 | b.反对 | c.同意 | d.完全同意 |
| **6.意外事件的发生会使我的身体一直承受风险** | a.完全反对 | b.反对 | c.同意 | d.完全同意 |
| **7.疼痛总是意味着身体收到了外界伤害** | a.完全反对 | b.反对 | c.同意 | d.完全同意 |
| **8.仅使疼痛加重的运动并不意味着它们很危险** | a.完全反对 | b.反对 | c.同意 | d.完全同意 |
| **9.我害怕会意外地伤害自己** | a.完全反对 | b.反对 | c.同意 | d.完全同意 |
| **10.目前我能做到的防止疼痛加剧最安全的事情就是不进行多余的运动，处处小心** | a.完全反对 | b.反对 | c.同意 | d.完全同意 |
| **11.如果我身体上没有潜在危险因素的话，我不会感到如此疼痛** | a.完全反对 | b.反对 | c.同意 | d.完全同意 |
| **12.我感到很痛，但如果我积极运动的话这种情况会有所好转** | a.完全反对 | b.反对 | c.同意 | d.完全同意 |
| **13.疼痛使我知道应何时停止运动以防止受伤** | a.完全反对 | b.反对 | c.同意 | d.完全同意 |
| **14.像我这样积极运动真的不安全** | a.完全反对 | b.反对 | c.同意 | d.完全同意 |
| **15.我太容易受伤了，无法做常人可以做的事** | a.完全反对 | b.反对 | c.同意 | d.完全同意 |
| **16.尽管有些事给我带来了疼痛，但我并不认为它们很危险** | a.完全反对 | b.反对 | c.同意 | d.完全同意 |
| **17.没有人在疼痛时必须要去运动** | a.完全反对 | b.反对 | c.同意 | d.完全同意 |

| **第四部分 对全膝关节置换围术期功能锻炼的行为实践**  总是：几乎100%能做到；经常：约能做到70%以上；有时：约能做到40-70%；很少：约能做到10-40%；从不：从来做不到或仅能做到10%以下 | | | | | |
| --- | --- | --- | --- | --- | --- |
| **1.我会主动了解全膝关节置换术后康复的相关知识。P** | a.总是 | b.经常 | c.有时 | d.很少 | e.从不 |
| **2.早期锻炼时有点痛感是正常的，我会继续坚持锻炼；但如果是剧烈疼痛，我会及时就医。P** | a.总是 | b.经常 | c.有时 | d.很少 | e.从不 |
| **3.术后恢复期间，我会积极参与医护人员指导下的功能锻炼，以促进膝关节康复。P** | a.总是 | b.经常 | c.有时 | d.很少 | e.从不 |
| **4.由于害怕，我可能回避或减少参与康复锻炼。N** | a.总是 | b.经常 | c.有时 | d.很少 | e.从不 |
| **5.为了改善膝关节活动度和肌肉力量，术后我会遵医嘱积极进行功能锻炼。P** | a.总是 | b.经常 | c.有时 | d.很少 | e.从不 |
| **6.我担心再次损伤膝关节，哪怕医生推荐我也不愿意进行较为剧烈的锻炼。N** | a.总是 | b.经常 | c.有时 | d.很少 | e.从不 |
| **7.我会积极参与康复锻炼，改善生活质量，减轻关节疼痛，更好地完成日常活动。P** | a.总是 | b.经常 | c.有时 | d.很少 | e.从不 |
| **8.我会积极与医护人员沟通，医护人员的支持对康复锻炼非常重要，他们的帮助能够让我更有信心应对康复过程。P** | a.总是 | b.经常 | c.有时 | d.很少 | e.从不 |
| **9.我会积极主动寻求康复锻炼的指导，以确保锻炼动作正确，避免不当动作造成伤害。P** | a.总是 | b.经常 | c.有时 | d.很少 | e.从不 |

| **再次感谢您参与填写我们的调查问卷，您回答所提供的信息对我们今后工作非常有价值！**  **Thank you for filling out our questionnaire！**  若您对本次调查研究有任何意见与建议，我们将十分荣幸能够聆听您的声音。  意见与建议： （选填）  为了本次问卷调研能够切实起到作用，推进今后回访工作的顺利开展，若您能够愿意留下联系方式，我们将不胜感激！  您的电话： （选填） |
| --- |
