# Supplementary material for: Knowledge attitude, and practice of patients with knee osteoarthritis towards perioperative functional exercise after total knee arthroplasty: a cross-sectional study
Source: PeerJ. 2025 Jun 4;13:e19511. doi: 10.7717/peerj.19511 (PMC12145091; doi:10.7717/peerj.19511)
Supplement: Supplemental Information 4 [file peerj-13-19511-s004.docx]

| Questionnaire ID: |
| --- |
| Dear Participant,  We are the Department of Joint Surgery at the Affiliated Hospital of Qingdao University, and we sincerely invite you to participate in our research study. This study aims to understand the **knowledge, attitudes, and practices of patients with knee osteoarthritis regarding perioperative functional exercises for total knee arthroplasty,** providing a basis for developing scientific intervention strategies. This may help more people in the future and improve their health conditions. Your participation in this study is voluntary, and the study has been reviewed and approved by the Ethics Review Committee. If you agree to participate, please refer to the following instructions:  1.Please complete the questionnaire. There are no right or wrong answers; you only need to fill it out based on your actual situation. If you have any questions during the process, feel free to ask us. Once completed, please submit it promptly.  2.This study is a simple questionnaire survey and will not cause any harm to your physical or mental health. However, it involves some personal information, such as your gender and age. We will strictly keep your information confidential and will not disclose it, so please feel assured when filling it out.  3.As a participant, you can access information related to this study and its progress at any time. If you decide to withdraw from the study, please inform us, and your data will not be included in the research results.  Finally, we sincerely appreciate your valuable time and support for our scientific research!  □I acknowledge and agree that the collected data will be used for scientific research.  Informed Consent Signature:  Participation Date: ______ (Year) ______ (Month) ______ (Day) |

| **Part 1 Basic Information** | | |
| --- | --- | --- |
| **1.Your gender:** | a. Male | b. Female |
| **2.Your age: ______** (Please enter a number; in Questionnaire Star, please enable numerical validation – integers only) | | |
| **3.Your place of residence:** | a. Rural b. Urban c. Suburban | |
| **4.Your education level:** | a. Junior high school or below  b. High school/Technical school  c. College  d. University  e. Master’s degree or above | |
| **5.Your occupation type:** | a. Government/agency officials/civil servants b. Professional technical personnel (e.g., teachers, doctors, engineers, writers) c. General staff and related personnel d. General workers (e.g., factory workers, manual laborers) e. Business/service industry personnel f. Self-employed/business contractors g. Freelancers h. Agricultural, forestry, animal husbandry, fishery, and water conservancy workers i. Production and transportation equipment operators and related personnel j. Retired k. Unemployed l. Other (please specify) | |
| **6.In the past year, your household’s average monthly income per person (including in-kind income and rental income):** | a.<2000  b.2000-5000  c.5000-10000  d.10000-20000  e.>20000 | |
| **7.Your marital status:** | a. Unmarried  b. Married c. Divorced d. Widowed | |
| **8.Do you have any underlying medical conditions? (Multiple selections allowed)** | a. Diabetes b. Hypertension c. Kidney disease d. Other (please specify) | |
| **9.Type of medical insurance:** | a. Only social medical insurance (e.g., employee medical insurance, "new rural cooperative medical care," "urban residents' basic medical insurance," etc.) b. Only commercial medical insurance c. Both social and commercial medical insurance d. No insurance | |
| **9.When were you diagnosed with knee osteoarthritis? (Please select a time)** | | |
| **10.Have you undergone total knee arthroplasty?** | a. Yes b. No | |

| **Part 2 Knowledge of Perioperative Functional Exercise for Total Knee Arthroplasty** | | | |  |
| --- | --- | --- | --- | --- |
| **Please indicate your level of understanding for the following statements:** | | | | |
| 1. **Through joint replacement, the pain and discomfort caused by degeneration of knee joint cartilage can be relieved. What is the key period for the recovery of knee joint function?**   The early postoperative period, especially within the first half month after surgery. | a. Very familiar | b. Heard of it | c. Not sure | |
| 1. **How well do you understand the benefits of active functional exercises before surgery?**   Preoperative active functional exercises can increase muscle strength, alleviate postoperative pain, and shorten the recovery time after surgery. | a. Very familiar | b. Heard of it | c. Not sure | |
| 1. **Are you aware of the benefits of early postoperative joint activity and functional exercises?**   Early postoperative joint activity and functional exercises can effectively reduce the occurrence of perioperative complications, facilitate the recovery of knee joint function and overall physical fitness. | a. Very familiar | b. Heard of it | c. Not sure | |
| 1. **Do you understand the purposes of rehabilitation exercises after knee joint replacement surgery?**   To improve and maintain normal joint mobility, enhance knee muscle strength, promote knee joint stability; to master joint protection techniques, avoid excessive pressure on the joint prosthesis, and extend the service life of the artificial joint as much as possible. | a. Very familiar | b. Heard of it | c. Not sure | |
| 1. **Do you know what rehabilitation exercises can be performed from the recovery of anesthesia after knee joint replacement surgery?**   You can perform muscle and distal joint rehabilitation exercises in bed, and on the first day after surgery, you can do knee joint flexion and extension exercises and get out of bed under the guidance of medical staff. | a. Very familiar | b. Heard of it | c. Not sure | |
| **6. Do you know how to perform functional exercises in bed?** | a. Very familiar | b. Heard of it | c. Not sure | |
| **7. Are you aware of the purpose of medical staff applying effective measures (painkillers, local cold compress, etc.) to control pain after surgery?**   - 1. To reduce pain and actively engage in rehabilitation training under the guidance of medical staff. | a. Very familiar | b. Heard of it | c. Not sure | |
| **8. Can rehabilitation exercises during the perioperative period of total knee arthroplasty be performed only on the affected limb?**   - 1. It is not just about exercising the affected limb, but should also include training for both upper limbs, the healthy side lower limb, and the affected limb. | a. Very familiar | b. Heard of it | c. Not sure | |
| **9. China's capital is Shanghai.** | a. True | b. False |  | |

| **Part 3 Attitudes Toward Perioperative Functional Exercise for Total Knee Arthroplasty** | | | | | |
| --- | --- | --- | --- | --- | --- |
| 1. I believe that early postoperative functional exercise after total knee arthroplasty is crucial for recovery and can significantly improve my quality of life. | a. strongly agree | b. agree | c. neutral | d. disagree | e. strongly disagree |
| 1. I consider it very dangerous to start functional exercises on the day of surgery, and I am very concerned even about activities in bed. | a. strongly agree | b. agree | c. neutral | d. disagree | e. strongly disagree |
| 1. I realize the importance of postoperative functional exercise for recovery, and I will strive to achieve the rehabilitation goals set before surgery. | a. strongly agree | b. agree | c. neutral | d. disagree | e. strongly disagree |
| 1. I do not understand the postoperative rehabilitation exercises for knee arthroplasty well and hope for more detailed guidance and explanation. | a. strongly agree | b. agree | c. neutral | d. disagree | e. strongly disagree |
| 1. Perioperative functional exercise for total knee arthroplasty involves not only the affected limb but also other parts. I am concerned about whether I can adapt to this exercise method. | a. strongly agree | b. agree | c. neutral | d. disagree | e. strongly disagree |
| 1. I understand that effective pain control is key to rehabilitation exercise. I will actively communicate with medical staff to ensure that the pain is effectively relieved. | a. strongly agree | b. agree | c. neutral | d. disagree | e. strongly disagree |

| **Part 4 Tampa Scale for Kinesiophobia (TSK)** | | | | |
| --- | --- | --- | --- | --- |
| 1. I am afraid that I will injure myself if I exercise at this time. | a. strongly disagree | b. disagree | c. agree | d. strongly agree |
| 1. If I try to overcome my fear, the pain will intensify. | a. strongly disagree | b. disagree | c. agree | d. strongly agree |
| 1. My body tells me that I have made a very serious mistake. | a. strongly disagree | b. disagree | c. agree | d. strongly agree |
| 1. If I exercise at this time, the pain is likely to be relieved. | a. strongly disagree | b. disagree | c. agree | d. strongly agree |
| 1. People around me do not pay enough attention to my health condition. | a. strongly disagree | b. disagree | c. agree | d. strongly agree |
| 1. Unexpected events will keep my body at risk. | a. strongly disagree | b. disagree | c. agree | d. strongly agree |
| 1. Pain always means that the body is being injured from the outside. | a. strongly disagree | b. disagree | c. agree | d. strongly agree |
| 1. Exercise that only worsens the pain does not necessarily mean it is dangerous. | a. strongly disagree | b. disagree | c. agree | d. strongly agree |
| 1. I am afraid of accidentally injuring myself. | a. strongly disagree | b. disagree | c. agree | d. strongly agree |
| 1. The safest thing I can do now to prevent the pain from worsening is to avoid excessive exercise and be cautious in everything I do. | a. strongly disagree | b. disagree | c. agree | d. strongly agree |
| 1. If I don't have any underlying risk factors, I wouldn't feel such pain. | a. strongly disagree | b. disagree | c. agree | d. strongly agree |
| 1. I am in a lot of pain, but if I exercise actively, this situation will improve. | a. strongly disagree | b. disagree | c. agree | d. strongly agree |
| 1. Pain tells me when to stop exercising to prevent injury. | a. strongly disagree | b. disagree | c. agree | d. strongly agree |
| 1. Being as active as I am really unsafe. | a. strongly disagree | b. disagree | c. agree | d. strongly agree |
| 1. I am too prone to injury to do what ordinary people can do. | a. strongly disagree | b. disagree | c. agree | d. strongly agree |
| 1. Although some things cause me pain, I don't think they are dangerous. | a. strongly disagree | b. disagree | c. agree | d. strongly agree |
| 1. No one has to exercise when they are in pain. | a. strongly disagree | b. disagree | c. agree | d. strongly agree |

| **Part 4 Behavioral Practice of Perioperative Functional Exercise for Total Knee Arthroplasty**  Always: almost 100% of the time; Often: about 70% or more of the time; Sometimes: about 40-70% of the time; Rarely: about 10-40% of the time; Never: almost never or less than 10% of the time | | | | | |
| --- | --- | --- | --- | --- | --- |
| 1. I will proactively seek knowledge about postoperative rehabilitation after total knee arthroplasty. | a. always | b. often | c. sometimes | d. rarely | e. never |
| 1. A slight discomfort during early exercise is normal, and I will continue to persevere. However, if there is severe pain, I will seek medical attention promptly. | a. always | b. often | c. sometimes | d. rarely | e. never |
| 1. During the recovery period after surgery, I will actively participate in functional exercises guided by medical staff to promote knee joint recovery. | a. always | b. often | c. sometimes | d. rarely | e. never |
| 1. Due to fear, I may avoid or reduce participation in rehabilitation exercises. | a. always | b. often | c. sometimes | d. rarely | e. never |
| 1. In order to improve knee joint mobility and muscle strength, I will actively engage in functional exercises as recommended by my doctor after surgery. | a. always | b. often | c. sometimes | d. rarely | e. never |
| 1. I am worried about injuring my knee joint again and am unwilling to engage in more vigorous exercises even if recommended by the doctor. | a. always | b. often | c. sometimes | d. rarely | e. never |
| 1. I will actively participate in rehabilitation exercises to improve my quality of life, reduce joint pain, and better perform daily activities. | a. always | b. often | c. sometimes | d. rarely | e. never |
| 1. I will actively communicate with medical staff. Their support is vital for rehabilitation exercises, and their assistance will give me more confidence in coping with the recovery process. | a. always | b. often | c. sometimes | d. rarely | e. never |
| 1. I will actively seek guidance for rehabilitation exercises to ensure correct movements and avoid improper actions that may cause injury. | a. always | b. often | c. sometimes | d. rarely | e. never |
